# Supplementary material for: Agavins Impact on Gastrointestinal Tolerability-Related Symptoms during a Five-Week Dose-Escalation Intervention in Lean and Obese Mexican Adults: Exploratory Randomized Clinical Trial
Source: Foods. 2022 Feb 24;11(5):670. doi: 10.3390/foods11050670 (PMC8909258; doi:10.3390/foods11050670)
Supplement: Supplementary file 1 [file foods-11-00670-s001.zip › foods-1577537-supplementary.pdf]

Table S1. Intra-group and against control comparisons of clinically relevant metabolic markers concentration among groups and doses during the five-week dose-escalation period in lean participants.

		Variable					
	Daily dose	GLU (mg/dL)	TRG (mg/dL)	CHOL-T (mg/dL)	HDL-C (mg/dL)	LDL-C (mg/dL)	VLDL-C (mg/dL)
AGAVINS	0 g	83.8 ± 2.5	157.1 ± 24.9	205.9 ± 14.6	50.66 ± 4.1	126.5 ± 13.7	31.47 ± 5
	2.5 g	82.13 ± 2.4	130.1 ± 20.1	197.5 ± 15.3	50.41 ± 4.8	124.1 ± 14.4	26 ± 4
	5 g	82.88 ± 2.3	130.1 ± 19.3	203.4 ± 11.6	51.86 ± 5.1	129.5 ± 11.2	26.10 ± 3.9
	7 g	84.25 ± 3.6	126.1 ± 15.1	204.5 ± 15.4	53.86 ± 5.3[b]	130.1 ± 15	25.20 ± 3
	10 g	80.89 ± 4.1	147.9 ± 21.4	200 ± 13	49.79 ± 4.6[b]	123.4 ± 13.5	29.56 ± 4.2
	12 g	84.33 ± 2.5	113.9 ± 14	202.4 ± 13.5	53.14 ± 3.9	130.8 ± 11.5	22.78 ± 2.8
PLACEBO	0 g	82 ± 3.2	105.8 ± 12.4	196.2 ± 12.4	51.84 ± 3.9	123.2 ± 9.3	21.17 ± 2.4
	2.5 g	83.28 ± 3.1	99.57 ± 18.4	195.8 ± 13.7	54.55 ± 4.8	121.3 ± 10.9	20 ± 3.6
	5 g	79.66 ± 3.6	93 ± 17.8	197.8 ± 18.8	53 ± 4.4	126.1 ± 14.5	18.66 ± 3.6
	7 g	84.28 ± 1.9	101.2 ± 16.7	195.7 ± 19.1	55.57 ± 5.5	119.8 ± 14.6	20.31 ± 3.3
	10 g	86.57 ± 1.4	95.28 ± 21	201 ± 18.1	53.32 ± 5.2	128.8 ± 13.8	30.42 ± 10.6
	12 g	81.28 ± 5.8	103.8 ± 25.4	196.2 ± 13.2	51.88 ± 2.6	123.6 ± 10.7	20.77 ± 5
	P value[a]	0.567	0.191	0.402	0.225	0.336	0.193

Abbreviations: CHOL-T, total cholesterol; GLU, fasting glucose; HDL-C, high-density lipoprotein cholesterol; LDL-C, low-density lipoprotein cholesterol; TRG, triglycerides; VLDL-C, very-low-density lipoprotein density. Data presented as mean ± SEM. [a]Obtained from a mixed-effects analysis with the Geisser-Greenhouse correction for agavins doses treatment. [b]Significant difference (P<.05) between 7 g/day and 10 g/day of agavins, Tukey's multiple comparisons test.

Table S2. Intra-group and against control comparisons of clinically relevant metabolic markers concentration among groups and doses during the five-week dose-escalation period in obese participants.

		Variable					
	Daily dose	GLU (mg/dL)	TRG (mg/dL)	CHOL-T (mg/dL)	HDL-C (mg/dL)	LDL-C (mg/dL)	VLDL-C (mg/dL)
AGAVINS	0 g	83.64 ± 4	164.5 ± 30.5	176.6 ± 12.1	41.45 ± 3.1	102.3 ± 8.1	32.93 ± 6.1
	2.5 g	84.82 ± 2.6	176.3 ± 28.4	177.4 ± 14.3	38.93 ± 2.7	103.1 ± 10.7	35.36 ± 5.6
	5 g	88.70 ± 3	219.7 ± 31.1[b]	181.9 ± 12.8	37.09 ± 2.4	100.8 ± 7.6	43.94 ± 6.2[d]
	7 g	80.91 ± 1.9	238 ± 35.9	182.8 ± 12.3	39.85 ± 2.5	95.34 ± 8.6	47.64 ± 7.1
	10 g	81.13 ± 3.1	145 ± 22.2	161.4 ± 6.6[c]	40.91 ± 2.6	91.55 ± 6.3	29.13 ± 4.4
	12 g	84.45 ± 3.1	195.7 ± 31.8	180.9 ± 11.9	40.71 ± 2.6	101.1 ± 8.4	39.15 ± 6.3
PLACEBO	0 g	85.27 ± 3.1	151.9 ± 20.1	173.7 ± 7.8	41.29 ± 3.1	102 ± 5.5	30.36 ± 4
	2.5 g	83 ± 2.5	136.1 ± 14	177.3 ± 7.3	44.25 ± 2.6	105.9 ± 6.6	27.04 ± 2.8
	5 g	85.18 ± 2.7	137.7 ± 11.8[b]	171.4 ± 6.3	43.35 ± 2.9	100.4 ± 5.7	27.54 ± 2.4[d]
	7 g	88.8 ± 4.6	177.6 ± 20	184.3 ± 5.2	43.85 ± 2	104.9 ± 5.6	35.52 ± 4
	10 g	85.09 ± 2.6	165.2 ± 23.3	183.1 ± 4.7[c]	45.67 ± 3.1	103.6 ± 3.4	33.09 ± 4.6
	12 g	83.45 ± 2.5	186.9 ± 22.7	178.8 ± 4.8	42.01 ± 2.8	99.41 ± 4.3	37.38 ± 4.5
	P value[a]	0.448	0.098	0.881	0.557	0.680	0.099

Abbreviations: CHOL-T, total cholesterol; GLU, fasting glucose; HDL-C, high-density lipoprotein cholesterol; LDL-C, low-density lipoprotein cholesterol; TRG, triglycerides; VLDL-C, very-low-density lipoprotein density. Data presented as mean ± SEM. [a]Obtained from a mixed-effects analysis with the Geisser-Greenhouse correction for agavins doses treatment. [b,c,d]Significant differences between agavins and placebo groups (P<.05) after the same dose, unpaired t test with Welch's correction.

Table S3. Body composition analysis during the five-week dose-escalation period in lean participants.

	Daily dose	BW (Kg)	RFM (Kg)	AFM (Kg)	FFM (Kg)	SMM (Kg)	TBW (L)	EW (L)
		Variable						
**AGAVINS**	0 g	60.58 ± 1.5	28.89 ± 2.2	17.42 ± 1.2	43.16 ± 1.9	19.46 ± 1.2	31.6 ± 1.3	13.03 ± 0.33
	2.5 g	60.66 ± 1.6	29.51 ± 2.5	17.83 ± 1.4	42.82 ± 2	19.29 ± 1.2	31.34 ± 1.3	12.93 ± 0.31
	5 g	60. 97 ± 1.9	29.29 ± 2.7	17.75 ± 1.5	43.22 ± 2.4	19.37 ± 1.4	31.66 ± 1.5	13.2 ± 0.41
	7 g	60.72 ± 1.7	29.99 ± 2.3	18.11 ± 1.3	42.61 ± 2.1	19.37 ± 1.2	31.28 ± 1.3	12.87 ± 0.33
	10 g	60.96 ± 1.6	29.94 ± 2.4	18.19 ± 1.4	42.76 ± 2	19.09 ± 1.2	31.27 ± 1.3	13.07 ± 0.31
	12 g	60.56 ± 1.6	29.57 ± 2.4	17.87 ± 1.4	42.69 ± 1.9	19.43 ± 1.1	31.3 ± 1.2	12.84 ± 0.34
**PLACEBO**	0 g	62.57 ± 3.8	24.38 ± 2.1	15.39 ± 1.7	47.15 ± 3	21.32 ± 1.9	34.56 ± 2.1	14.34 ± 0.92
	2.5 g	62.21 ± 3.9	23.97 ± 2.0	15.08 ± 1.6	47.26 ± 3.1	21.6 ± 2	34.78 ± 2.3	14.32 ± 0.92
	5 g	63.01 ± 4.5	23.24 ± 1.5	14.89± 1.9	48.29 ± 3.3	22.02 ± 2	35.32 ± 2.3	14.54 ± 1
	7 g	62.61 ± 3.9	24.06 ± 2.2	15.16 ± 1.5	47.48 ± 3	21.23 ± 1.9	34.73 ± 2.2	14.56 ± 0.93
	10 g	62.19 ± 3.9	24.38 ± 2.2	15.36 ± 1.6	47.15 ± 2.9	20.97 ± 1.9	34.24 ± 2.2	14.32 ± 0.89
	12 g	61.74 ± 4.3	23.7 ± 1.9	14.78 ± 1.5	48.36 ± 3.2	21.11 ± 1.9	34.34 ± 2.3	14.29 ± 1
	P value[a]	0.123	0.114	0.153	0.265	0.156	0.197	0.123

Abbreviations: BW, body weight; RFM, relative fat mass; AFM, absolute fat mass; FFM, fat-free mass; SMM, skeletal muscle mass; TBW, total body water; EW, extracellular water. [a]Obtained from a mixed-effects analysis with the Geisser-Greenhouse correction for agavins dose treatment. No significant differences (*P*<.05) for intra-group comparisons in agavins group by Holm-Šidák's multiple comparisons test, nor for comparisons against control for each dose by an unpaired t test with Welch's correction.

Table S4. Body composition analysis during the five-week dose-escalation period in obese participants.

	Daily dose	BW (Kg)	RFM (Kg)	AFM (Kg)	FFM (Kg)	SMM (Kg)	TBW (L)	EW (L)
		Variable						
**AGAVINS**	0 g	88.43 ± 3.5[b]	43.13 ± 1.9	38.16 ± 2.3	50.27 ± 2.7	23.27 ± 1.6	37.27 ± 2	16.41 ± 0.68
	2.5 g	88.23 ± 3.7	43.66 ± 1.8	38.58 ± 2.4	49.65 ± 2.6	23.24 ± 1.5	36.83 ± 1.9	16 ± 0.69
	5 g	89.72 ± 3.6	43.35 ± 2	38.89 ± 2.4	50.82 ± 2.8	23.74 ± 1.6	37.68 ± 2	16.44 ± 0.74
	7 g	88.21 ± 3.5	43.36 ± 1.9	38.28 ± 2.4	49.93 ± 2.7	23.15 ± 1.5	36.99 ± 1.9	16.25 ± 0.72
	10 g	85.07 ± 4[b]	42.79 ± 2.4	36.61 ± 3	48.46 ± 2.7	22.55 ± 1.5	35.92 ± 1.9	15.62 ± 0.72
	12 g	87.44 ± 3.9	42.46 ± 2.2	37.28 ± 2.7	50.16 ± 2.8	23.52 ± 1.6	37.2 ± 2	16.11 ± 0.77
**PLACEBO**	0 g	85.46 ± 4	39.59 ± 2.2	33.85 ± 2.7	51.61 ± 2.9	23.74 ± 1.8	38.16 ± 2.2	16.69 ± 0.8
	2.5 g	84.48 ± 4.1	39.83 ± 2.4	33.66 ± 2.8	50.82 ± 3.1	23.3 ± 1.8	37.61 ± 2.3	16.44 ± 0.82
	5 g	85.25 ± 3.8	39.36 ± 2.4	33.51 ± 2.7	51.74 ± 3.1	23.66 ± 1.8	38.19 ± 2.3	16.81 ± 0.83
	7 g	84.27 ± 3.9	38.76 ± 2.6	32.56 ± 2.7	51.71 ± 3.3	23.63 ± 2	38.07 ± 2.4	16.62 ± 0.8
	10 g	85.19 ± 3.7	39.34 ± 2.5	33.45 ± 2.6	51.74 ± 3.1	23.77 ± 1.8	38.23 ± 2.3	16.76 ± 0.79
	12 g	85.24 ± 3.7	39.27 ± 2.5	33.44 ± 2.7	51.8 ± 3.1	23.7 ± 1.8	38.24 ± 2.3	16.88 ± 0.78
	P value[a]							

Abbreviations: BW, body weight; RFM, relative fat mass; AFM, absolute fat mass; FFM, fat-free mass; SMM, skeletal muscle mass; TBW, total body water; EW, extracellular water. [a]Obtained from a mixed-effects analysis with the Geisser-Greenhouse correction for agavins dose treatment. [b]Significant difference (*P*<.05) between 0 and 10 g/day of agavins, Holm-Šidák's multiple comparisons test.
